# CLAIRE: contrastive learning-based batch correction framework for better balance between batch mixing and preservation of cellular heterogeneity

**DOI:** 10.1093/bioinformatics/btad099

**Published:** 2023-02-23

**Authors:** Xuhua Yan, Ruiqing Zheng, Fangxiang Wu, Min Li

**Affiliations:** Hunan Provincial Key Lab on Bioinformatics, School of Computer Science and Engineering, Central South University, Changsha 410083, China; Hunan Provincial Key Lab on Bioinformatics, School of Computer Science and Engineering, Central South University, Changsha 410083, China; Division of Biomedical Engineering, Department of Computer Science, Department of Mechanical Engineering, University of Saskatchewan, Saskatoon, SK S7N 5A9, Canada; Hunan Provincial Key Lab on Bioinformatics, School of Computer Science and Engineering, Central South University, Changsha 410083, China

## Abstract

**Motivation:**

Integration of growing single-cell RNA sequencing datasets helps better understand cellular identity and function. The major challenge for integration is removing batch effects while preserving biological heterogeneities. Advances in contrastive learning have inspired several contrastive learning-based batch correction methods. However, existing contrastive-learning-based methods exhibit noticeable *ad hoc* trade-off between batch mixing and preservation of cellular heterogeneities (mix-heterogeneity trade-off). Therefore, a deliberate mix-heterogeneity trade-off is expected to yield considerable improvements in scRNA-seq dataset integration.

**Results:**

We develop a novel contrastive learning-based batch correction framework, CIAIRE, which achieves superior mix-heterogeneity trade-off. The key contributions of CLAIRE are proposal of two complementary strategies: construction strategy and refinement strategy, to improve the appropriateness of positive pairs. Construction strategy dynamically generates positive pairs by augmenting inter-batch mutual nearest neighbors (MNN) with intra-batch k-nearest neighbors (KNN), which improves the coverage of positive pairs for the whole distribution of shared cell types between batches. Refinement strategy aims to automatically reduce the potential false positive pairs from the construction strategy, which resorts to the memory effect of deep neural networks. We demonstrate that CLAIRE possesses superior mix-heterogeneity trade-off over existing contrastive learning-based methods. Benchmark results on six real datasets also show that CLAIRE achieves the best integration performance against eight state-of-the-art methods. Finally, comprehensive experiments are conducted to validate the effectiveness of CLAIRE.

**Availability and implementation:**

The source code and data used in this study can be found in https://github.com/CSUBioGroup/CLAIRE-release.

**Supplementary information:**

Supplementary data are available at *Bioinformatics* online.

## 1 Introduction

Single-cell RNA sequencing (scRNA-seq) was developed to characterize high-throughput gene expression profiles for populations of individual cells, which has enabled an unprecedented resolution of cellular heterogeneity in complex tissues and has profoundly changed our understandings of cell-to-cell heterogeneity in various biological areas (Cao and Gao, 2022; Heath *et al.*, 2016; Lawson *et al.*, 2018; Tabula Muris Consortium *et al.*, 2018). Widespread adoption of scRNA-seq has produced a number of datasets. The integration of scRNA-seq datasets from multiple sources is critical for deciphering cellular heterogeneity in complex biological systems (Wang *et al.*, 2021). However, inherent technical differences among datasets caused by different experimental batches, sample donors or platforms lead to inevitable batch effects which can confound the biological variations (Luecken *et al.*, 2022; Tran *et al.*, 2020; Zheng *et al.*, 2019). Therefore, it’s vital to develop computational methods to correct batch effects.

Limma (Smyth and Speed, 2003) and ComBat (Johnson *et al.*, 2007) that were developed for bulk datasets were first applied to integrate scRNA-seq datasets. However, due to heterogeneous composition of cell populations between datasets and technical noise such as ‘dropout’ events (Chen *et al.*, 2022), Limma and ComBat were proven insufficient for single-cell datasets. To handle data with such characteristics, a number of batch correction methods have been proposed recently. A typical class of methods, such as MNNCorrect (Haghverdi *et al.*, 2018), Seurat (Stuart *et al.*, 2019), Scanorama (Hie *et al.*, 2019), use mutual nearest neighbors (MNN) between batches as anchors to map one dataset to another. To improve the effectiveness of MNN-based methods, some researchers propose to take cluster information into consideration, which cluster each batch first and then find MNN between clusters, such as scMerge (Lin *et al.*, 2019) and sMNN (Yang *et al.*, 2021c). Similarly, Harmony (Korsunsky *et al.*, 2019) employs soft clustering to maximize mixture of batches within clusters. With the increasing number of scRNA-seq datasets, the application of deep learning techniques, especially unsupervised ones, has received greater attention in this field. MMD-ResNet (Shaham *et al.*, 2017) assumes that the difference between the whole distributions of two batches is moderate and then trains a residual network with maximum mean discrepancy (MMD) loss to learn a map from one distribution to another. Bermuda (Wang *et al.*, 2019) also adopts the MMD loss, but it optimizes loss at the cluster level instead of the whole batch. To strengthen the expressiveness of traditional autoencoders, iMAP (Wang *et al.*, 2021) proposes a novel deep learning framework by combining the power of autoencoders and generative adversarial networks.

Recently, contrastive learning (CL) has shown striking advantages in various domains (Wei *et al.*, 2021; Zeng *et al.*, 2021). Some CL-based batch correction methods have also been proposed. CL learns representations by concentrating positive pairs and separating negative pairs (Chen *et al.*, 2020). The basic idea behind CL-based batch correction methods is to construct inter-batch positive cell pairs with similar transcription and negative cell pairs with dissimilar transcription. Then, they employ a contrastive loss to concentrate positive pairs and separate negative pairs, thereby mitigating batch effects. For instance, INSCT (Simon *et al.*, 2021) and MAT^2^ (Zhang *et al.*, 2021) find inter-batch MNN as positive pairs and use random sampling to construct negative pairs. In addition, INSCT applies within-batch k-nearest neighbors (KNN) to complete positive samples for those cells without MNN. Then, INSCT and MAT^2^ optimize with triplet loss (Chen *et al.*, 2020). SMILE (Xu *et al.*, 2022) and CLEAR (Han *et al.*, 2021) employ random augmentations to generate positive pairs and use random sampling to construct negative pairs, and then optimize with InfoNCE loss (van den Oord *et al.*, 2018). In general, positive pairs play an important role in the performance of CL methods (Grill *et al.*, 2020; Tian *et al.*, 2020) and they basically determine the degree of batch effect removal. More positive pairs can better cover shared populations between batches but may introduce more false positive pairs (pairs of cells with different types) without the help of cell type annotations, implying better batch mixing but tending to over-correct. In contrast, inadequate positive pairs can guarantee the correctness of positive pairs but poorly cover the shared populations between batches, implying better preservation of cellular heterogeneity but compromising to batch mixing. However, it’s hard to determine ideal positive pairs in different situations and thus, CL-based methods have to make a trade-off between batch correction and preserving heterogeneity. We call this problem the mix-heterogeneity trade-off and find that most of existing CL-based methods suffer from this trade-off.

To achieve better mix-heterogeneity trade-off, we propose a novel CL-based batch correction framework with AutomatIc label REfinement (CLAIRE). The key contributions of CLAIRE are proposal of two complementary strategies to ensure the appropriate positive pairs during learning process. The first strategy dynamically generates positive pairs by augmenting inter-batch MNN using intra-batch KNN, which greatly improves the diversity of positive pairs and promotes better batch correction. The second strategy is proposed to remove the potential false positive pairs by resorting to the memorization effect of deep neural networks, which improves the correctness of generated positive pairs and promotes better preservation of cellular heterogeneity. These two effective strategies help CLAIRE achieve superior mix-heterogeneity trade-off over existing CL-based batch correction methods. Benchmarking results on six real datasets also show that CLAIRE achieves the best integration performance and has comparable computational consumptions to other methods. We further conduct comprehensive ablation experiments to validate the effectiveness of our proposed method.

## 2 Materials and methods

### 2.1 Overview

**Fig. 1.**
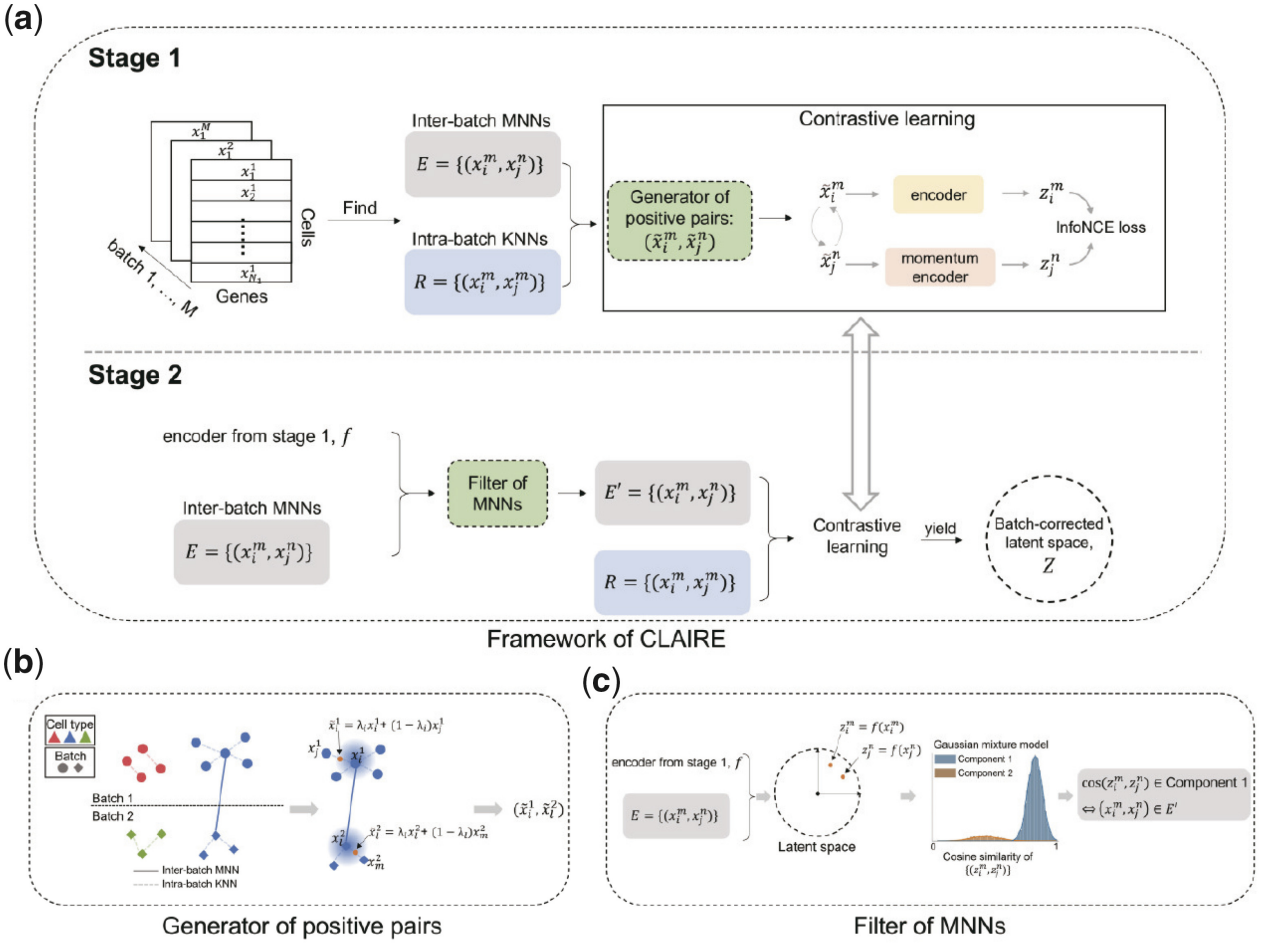
Architecture of CLARIE. (**a**) CLAIRE’s framework. Cells of multiple batches are projecting into a batch-corrected latent space in two stages. (**b**) Our proposed construction strategy for positive pairs, corresponding to the **generator** in (a). (**c**) Our proposed refinement strategy, corresponding to the **filter** in (a)

CLAIRE projects multiple scRNA-seq datasets into a low-dimensional space, as shown in Figure 1. Before CL, CLAIRE computes MNN between batches and KNN within each batch. In CL, CLAIRE uses these inter-batch MNN pairs as seeds of positive pairs and augments these seeds with intra-batch KNN to generate positive pairs (Fig. 1b, construction strategy). During the learning process, to reduce potential false positive pairs, CLAIRE divides model training into two stages. In the first stage, CLAIRE trains a neural network adapted from Moco (He *et al.*, 2020) architecture. Then, CLAIRE exploits the embeddings from the neural network to filter those MNNs that could introduce false positive pairs (Fig. 1c, refinement strategy). The retained MNNs are used for the second stage training. In the following sections, we elaborate on the detail of CLAIRE.

### 2.2 Dynamic construction of positive pairs

Suppose there are *M* batches to be integrated, $B_{m}={\left\{x_{i}^{m}\right\}}_{i=1}^{N(m)}(m\in \{1,2,\ldots ,M\})$, where $x_{i}^{m}$ denotes the preprocessed cell representations within batch *m*. $E_{mn}=\{(x_{i}^{m},x_{j}^{n})\}$ and $R_{m}=\{(x_{i}^{m},x_{j}^{m})\}$ respectively denote inter-batch MNNs between batch *m*, *n* and intra-batch KNNs within batch *m*. The parameter $K_{r}$ defines the number of nearest neighbors for searching intra-batch KNNs. Following Seurat (Stuart *et al.*, 2019), before finding MNNs between batches, CLAIRE applies canonical correlation analysis to map cells into low-dimensional representations. Those found inter-batch MNNs, *E*, are regarded as seeds of positive pairs. One potential problem of directly feeding these seed pairs into training is that they may not fully cover the whole distributions of the shared cell types between batches (Wang *et al.*, 2021). Hence, CLAIRE proposes using the intra-batch KNNs to augment those seed pairs. Specifically, for each seed pair $\left(x_{i}^{m},x_{j}^{n}\right)$, CLAIRE mix up cell $x_{i}^{m},x_{j}^{n}$ with their KNNs, respectively:


(1) $$\begin{array}{c} {\tilde{x}}_{i}^{m}=\lambda_{i}x_{i}^{m}+\left(1-\lambda_{i}\right)x_{k}^{m} \end{array},$$

(2) $${\tilde{x}}_{j}^{n}=\lambda_{j}x_{j}^{n}+\left(1-\lambda_{j}\right)x_{l}^{n},$$

where $(x_{i}^{m},x_{k}^{m})\in R_{m},(x_{j}^{n},x_{l}^{n})\in R_{n},\lambda_{i},\lambda_{j}$ are dynamically sampled from a uniform distribution U(α,1) and α is a positive constant <1. Note that for each generation, $\lambda_{i},\lambda_{j}$ are re-sampled from the uniform distribution. By such augmentation, more diverse positive pairs $\left({\tilde{x}}_{i}^{m},{\tilde{x}}_{j}^{n}\right)$ can be derived from original seed pairs. Essentially, CLAIRE derives two local regions from each seed pair, and turns the alignment of inter-batch MNNs into alignment of local regions between batches, thereby expanding the coverage of positive pairs for the distribution of shared populations between batches. Moreover, such generation can be implemented dynamically during model training, which requires little computational overhead. Except interpolation, other operations for mixing up cells with their KNNs can also be applied to augment the positive pairs, such as exchanging elements of two vectors. Apart from positive pairs, our CL framework needs negative pairs for training. Following other CL-based methods, CLAIRE randomly samples cells from the whole dataset to generate negative samples for each positive pair. Although random sampling can result in false negative pairs, the number of false negative pairs is supposed to be small because the number of negative samples for each cell is much larger than that of positive samples. Those rare false negative pairs have small impact on the results.

### 2.3 Automatic refinement of positive pairs

Instead of refining those generated positive pairs, we focus more on refining the seeds of positive pairs, which is the main source of false positive pairs. True seeds (MNNs with same cell type) generally lead to true positive pairs while false seeds (MNNs with different cell types) generally lead to false positive pairs. Thus, our goal here is to discriminate between true seeds and false seeds. According to the assumptions of MNNCorrect, the batch-effect variation is much smaller than the biological-effect variation between different cell types (Haghverdi *et al.*, 2018). A natural idea is to calculate the similarities between each pair of seed using raw expressions and MNNCorrect’s assumptions assure that the similarities between true seeds are much higher than false ones. Then, we can filter those false seeds with low similarities. However, in real situations, differences between batch-effect variation and biological-effect variation are not always distinct (Yang *et al.*, 2021b), which means that it’s not easy to directly discriminate between true seeds and false seeds. Therefore, our goal becomes to distinct false seeds from the true ones. Fortunately, Yang *et al.* (2021a) have found that true negative pairs are easier to optimize than false (noisy) negative pairs in the early training stage of CL, which is believed to be caused by the memorization effect of deep neural network (Arpit *et al.*, 2017), i.e. deep neural network tends to prioritize learning simple patterns first, and they exploit this finding to distinct false negative pairs from true negative ones.

Motivated by finding in Yang *et al.* (2021a) and MNNCorrect’s assumptions, CLAIRE proposes to use CL to amplify the difference between true seeds and false seeds. Specially, CLAIRE assumes that true seeds and false seeds have different patterns, and true seeds account for the majority of seeds and have simpler patterns (e.g. smaller distance or more consistent orientation) than false seeds. Then, true seeds (and their generations) are easier to fit than false ones (and their generations) in the early stage of CL. In other words, after early stage of training, the latent representations’ similarities between true seeds are supposed to be higher than false ones, which provides the foundation for removing false seeds.

Formally, CLAIRE divides model training into two stages. In the first stage, CLAIRE trains an encoder network, *f*, in a few epochs (e.g. 2–4 epochs). The encoder network, *f*, embeds samples $X=[x_{1},\ldots ,x_{s}]$ into latent representations $Z=[z_{1},\ldots ,z_{s}]$. Then, CLAIRE computes cosine similarities between each seed pair in the latent space:


(3) $$S\left(z_{i}^{m},z_{j}^{n}\right)=\frac{\langle z_{i}^{m},z_{j}^{n}\rangle }{\left\|z_{i}^{m}\right\|\cdot \left\|z_{j}^{n}\right\|},$$

where $z_{i}^{m}=f\left(x_{i}^{m}\right),z_{j}^{n}=f\left(x_{j}^{n}\right),\left(x_{i}^{m},x_{j}^{n}\right)\in E_{mn},\langle z_{i}^{m},z_{j}^{n}\rangle$ denotes inner produce of two vectors and $\left||z_{i}^{m}|\right|$ denotes the $L_{2}$-norm of $z_{i}^{m}$. CLAIRE applies a two-component Gaussian Mixture Model (GMM) to fit the similarity distribution:


(4) $$p(S)=\sum_{k=1}^{K_{g}=2}\gamma_{k}\varphi (S|k),$$

where $\gamma_{k}$ and φ(S|k) are the mixture coefficients and probability density of the *k*-th component, respectively. After fitting the GMM with maximum likelihood estimations, the confidence of each seed belonging to true seeds can be inferred:


(5) $$c\left(\left(x_{i}^{m},x_{j}^{n}\right)\right)=p\left(k|S\left(z_{i}^{m},z_{j}^{n}\right)\right),$$

where *k* denotes the true seed’s corresponding component. We select the component with larger mean of similarity scores. By setting a threshold η, CLAIRE filters those pairs with low confidence. The rest seeds are fed into the second stage training.

### 2.4 Network architecture and loss function

CLAIRE’s network architecture is adapted from Moco, which contains an online encoder, *f*, and a momentum encoder, *g*. *f* and *g* share the same structure. Given *N* positive pairs ${\left\{\left({\tilde{x}}_{2i-1},{\tilde{x}}_{2i}\right)\right\}}_{i=1}^{N}$ at each iteration, where $\left({\tilde{x}}_{2i-1},{\tilde{x}}_{2i}\right)$ denotes a dynamically generated positive pair. CLAIRE optimizes the following InfoNCE loss:


(6) $$\begin{array}{c} L=-\frac{1}{N}\sum_{i=1}^{N}\;\text{log}\;\left(\frac{\sigma \left({\tilde{x}}_{2i-1},{\tilde{x}}_{2i}\right)}{\sigma \left({\tilde{x}}_{2i-1},{\tilde{x}}_{2i}\right)+\sum_{q_{j}\in D}\sigma \left({\tilde{x}}_{2i-1},q_{j}\right)}\right)\\ +\text{log}\;\left(\frac{\sigma \left({\tilde{x}}_{2i},{\tilde{x}}_{2i-1}\right)}{\sigma \left({\tilde{x}}_{2i},{\tilde{x}}_{2i-1}\right)+\sum_{q_{j}\in D}\sigma \left({\tilde{x}}_{2i},q_{j}\right)}\right) \end{array},$$

where $\sigma \left({\tilde{x}}_{2i-1},{\tilde{x}}_{2i}\right)=\text{exp}\;\left(\frac{\langle f\left({\tilde{x}}_{2i-1}\right),g\left({\tilde{x}}_{2i}\right)\rangle }{\tau {\left\|f\left({\tilde{x}}_{2i-1}\right)\right\|}_{2}\cdot {\left\|g\left({\tilde{x}}_{2i}\right)\right\|}_{2}}\right)$ with a constant parameter τ. The dictionary $D={\left\{q_{i}\right\}}_{i=1}^{Q}$ denotes a memory queue which is used to save negative keys and is often of large size. By fixing *g* and updating *f* in equation, CLAIRE pushes positive pairs closer in the latent space while pushing each sample away from its negative keys in dictionary *D*. For *g*, it’s updated via exponential moving average, i.e. g=(1−ϵ)g+ϵf with a small constant ϵ∈(0,1). After each iteration, *D* is updated by the mini-batch features ${\left\{g\left({\tilde{x}}_{i}\right)\right\}}_{i=1}^{2N}$ in a first-in and first-out order. The main difference between original Moco architecture and CLAIRE is the way of aligning the positive samples, which is reflected in two aspects. First, CLAIRE inputs positive pairs into model symmetrically. More specifically, Moco inputs the anchor cell to *f* and inputs its positive to *g* while CLAIRE not only inputs the anchor to *f*, positive to *g* but also inputs positive to *f*, anchor to *g*. Second, CLAIRE’s loss function will align the anchor to positive and also align the positive to anchor whereas Moco only aligns the anchor to positive.

### 2.5 Implementation details

The encoder network in CLAIRE consists of three fully connected layers and one L2-normalization layer. Its input sizes are equal to the number of input genes while the output size is set to 128 by default. ReLU (Glorot *et al.*, 2011) is used as the activation function for the hidden layer. Dropout (Srivastava *et al.*, 2014) layer is used after each hidden layer during training and discarded during inference. The dropout rate is set to 0.3. The proposed CL framework is implemented with Pytorch and trained with Adam (Kingma and Ba, 2014) with initial learning rate 1e−4. During training, the uniform distribution parameter α is set to 0.5 and $K_{r}$ is set to 10. The minibatch size, *N*, is set to 256. τ is set to 0.1. ϵ is set to 0.001. Dictionary size, *Q*, is set to 2048.

## 3 Results

### 3.1 Datasets and preprocessing

We collect six real datasets for experiments. These datasets cover different integration tasks, including integration across samples and across platforms (10×, Drop-seq, and SMART-seq, etc.), separation of cell subtypes, integration of two batches or multiple batches. They also cover diverse cell types and different species, such as mouse cells, human lung cells and human immune cells. The cell type annotations and batch labels of these datasets are known in advance. The details for six real datasets are shown in Table 1.

**Table 1.** Details of six real datasets

Dataset	Platform	Number of batches	Number of cells	Number of cell types	Number of shared types	References
MCA	Microwell-seq, Smart-Seq2	2	6954	11	11	Tran *et al.* (2020)
PBMC	10X 3′, 10X 5′	2	15 476	9	9	Tran *et al.* (2020)
Pancreas	inDrop, CEL-Seq2, Smart-Seq2, SMARTer, SMARTer	5	14 767	15	4	Tran *et al.* (2020)
Immune (human)	10X, Smart-Seq2	10	33 506	16	2	Luecken *et al.* (2022)
Lung	10X, Drop-seq	16	32 472	17	2	Luecken *et al.* (2022)
Muris	Droplet, FACS	2	67 354	28	26	Yan *et al.* (2022)

Six datasets are preprocessed in the following steps. First, low informative genes expressed in fewer than three cells are removed. Then, the total counts of each cell are normalized to 10 000 followed by log-transformation. After normalization, highly variable genes (HVGs) are selected for each dataset through the dispersion-based method (Satija *et al.*, 2015). By default, top 2000 HVGs are selected for each dataset. For Muris dataset (the largest one), top 5000 HVGs are used to have better preservation of cellular heterogeneity.

### 3.2 Evaluation metrics

We use average silhouette width with batch labels (bASW) (Luecken *et al.*, 2022) and k-nearest-neighbor Batch-Effect Test (kBET) (Luecken *et al.*, 2022) to evaluate the performance of batch correction methods on batch mixing. bASW is obtained by calculating the silhouette width with batch labels for each cell type and averaging over all cell types. bASW describes mixing of batches within cell clusters, where 1 indicates ideally mixed batches and 0 indicates poorly mixed batches(Luecken *et al.*, 2022). kBET measures how well mixed the batches are, which is calculated based on the local batch label distribution in randomly sampled nearest-neighbor cells compared against the global batch label distribution (Zhao *et al.*, 2021). kBET is calculated as in Zhao *et al.* (2021), and the k is set to 15. Higher kBET indicates better batch effect removal.

Adjusted rand index (ARI) and normalized mutual information (NMI) are employed to evaluate the performance of batch correction methods on preserving cellular heterogeneity. NMI and ARI compare the overlap between clustering results and annotated labels (Liang *et al.*, 2021; Tian *et al.*, 2021). Higher NMI and ARI indicate better match between clustering results and annotations. Louvain is applied to determine the clusters with increased resolutions from 0.1 to 2.0 at the increment of 0.1. The clustering output with the highest NMI is chosen as the final clustering result. To better demonstrate the performance of batch correction methods on batch mixing and preserving heterogeneity, four metrics are aggregated into two scores: $S_{\text{bio}}$ and $S_{\text{batch}}$. For dataset *i*, two scores are calculated via:


(7) $$\begin{array}{cc} S_{\text{bio}}= & \frac{{\text{NMI}}_{i}+{\text{ARI}}_{i}}{2} \end{array}.$$

(8) $$S_{\text{batch}}=\frac{{\text{bASW}}_{i}+{\text{kBET}}_{i}}{2}.$$

Following Luecken *et al.* (2022), each metric is min–max scaled before metrics aggregation so that all metrics have equal weights. Then two scores are integrated by calculating a *F*1-score (batch correction) as follows:


(9) $$F1_{bc}=2\frac{S_{\text{bio}}*S_{\text{batch}}}{\left(S_{\text{bio}}+S_{\text{batch}}\right)}.$$

### 3.3 CLAIRE achieves superior mix-heterogeneity trade-off over other CL-based batch correction methods

**Fig. 2.**
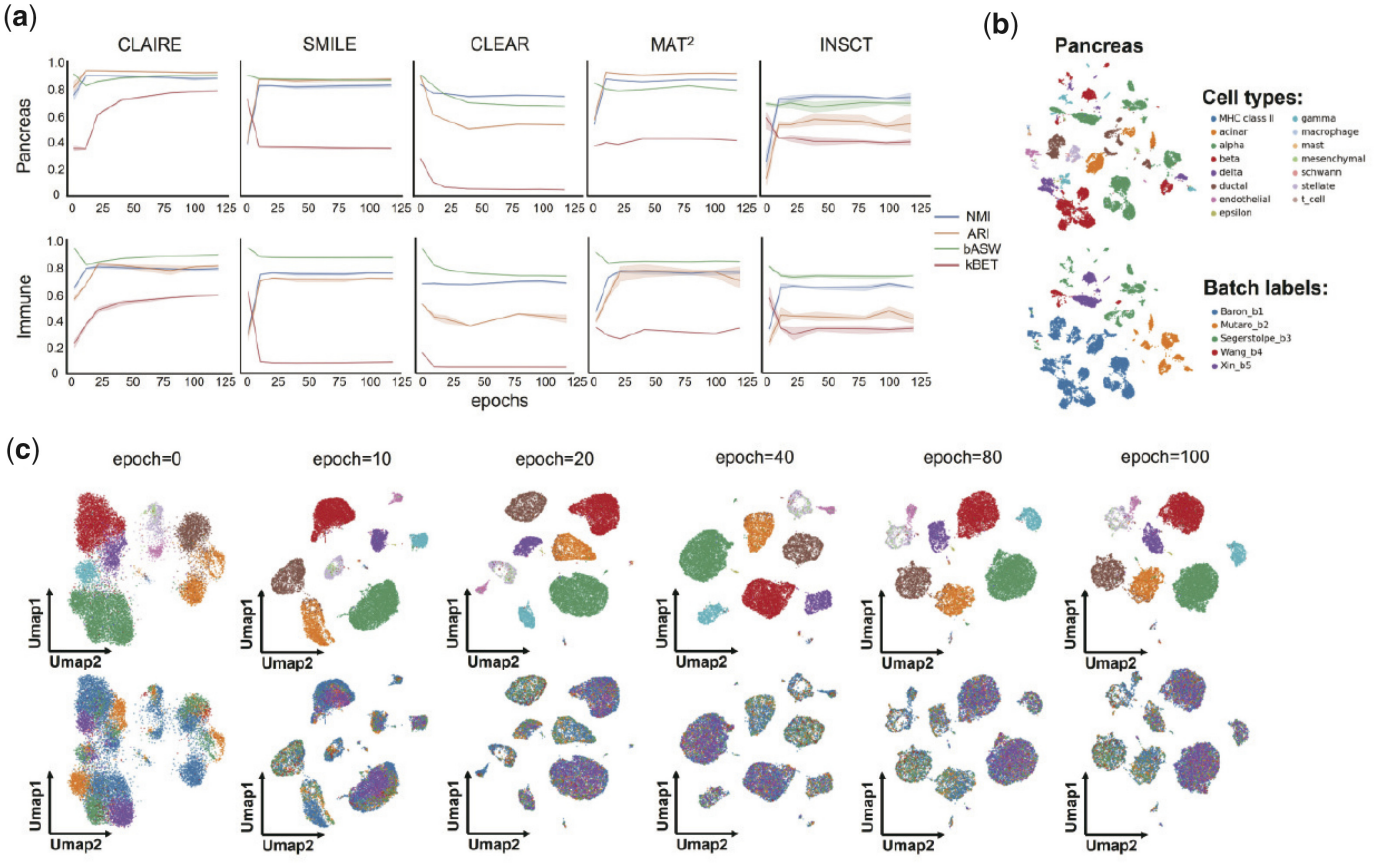
Mix-heterogeneity trade-off of CL-based batch correction methods. (**a**) Curves of four evaluation metrics with training epochs from CLAIRE, SMILE, CLEAR, MAT^2^, INSCT on Pancreas and Immune datasets. (**b**) UMAP visualizations of raw expressions of Pancreas dataset. Cells in the first row are colored by cell type annotations, and colored by batch labels in the second row. (**c**) UMAP visualizations of CLAIRE’s embeddings at different training epochs. Cells are colored by cell type in the first row and colored by batch labels in the second row. The cell color markers are consistent with b

To study the mix-heterogeneity trade-off throughout the training process, we plot CLAIRE’s bASW, kBET, ARI and NMI curves with training epochs on Pancreas, Immune and Lung datasets. Curves of other CL-based methods including INSCT, MAT^2^, SMILE and CLEAR are also plotted. Settings for these competing methods are shown in Supplementary Note S1. All methods are trained until the loss plateaus or decreasing quantity below 0.01, and they are run five times on each dataset. Results are shown in Figure 2a and Supplementary Figure S1. From the results after convergence, CLAIRE shows superior balance between batch mixing scores and heterogeneity preservation scores. In particular, CLAIRE distinctly outperforms other methods with respect to batch mixing scores on Pancreas and Immune datasets, while achieving competitive heterogeneity preservation scores on three datasets. From the convergence process, although CLAIRE’s kBET has a low start point but it greatly benefits from training and rises throughout the training process. Similar to CLAIRE, CLEAR and MAT^2^ also have a low start point of kBET but MAT^2^’s kBET does not benefit from training while CLEAR’s kBET even decreases with training, which implies that CLAIRE’s construction strategy of positive pairs provides stronger integration target. What’s more, even though CLAIRE mixes batches better as training proceeds, its heterogeneity preservation scores do not drop from the peak values. Inspired by this finding, it can be interpretted that CLAIRE first concentrates cells with the same type from a global standpoint, and then mixes batches within local cell clusters.

To explain above results more intuitively, we plot all methods’ outputs at different epochs on Pancreas dataset using Uniform Manifold Approximation and Projection (UMAP) (Becht *et al.*, 2019). Figure 2b shows the visualizations of raw expressions without integration. Figure 2c and Supplementary Figure S2 show the visualizations of CLARIE and other methods, respectively. It can be observed from these figures that different methods have distinct embeddings at epoch 0. This is caused by many factors including different model architectures, different weight initialization, and different preprocessing steps. In Figure 2c, cell clusters at 0 epoch are clearly separated due to batch effects, but with epoch increasing, CLAIRE mixes batches more sufficiently while cell clusters of different types remain clearly separated to each other, which is consistent with CLAIRE’s metric curves. In Supplementary Figure S2b, cells are closely distributed in SMILE’s embedding space at 0 epoch. Thus, batch effect is small and cellular heterogeneity is also low, which explains SMILE high kBET/bASW and low ARI/NMI at initial training stage. With epoch increasing, boundaries between cells clusters of different types become clearer while batches become less sufficiently mixed than beginning, which explains SMILE’s increasing ARI/NMI and decreasing kBET/bASW. The similar phenomena can also be found in INSCT and MAT^2^’s results. For CLEAR, it’s observed that the batch mixing results become worse as training proceeds and its cellular heterogeneity becomes more ambiguous. That might be because CLEAR’s construction strategy of positive pairs defines an unreasonable integration target. From these results, we can conclude that CLAIRE realizes remarkable improvement over existing CL-based batch correction methods with respect to mix-heterogeneity trade-off.

### 3.4 Benchmarking of CLAIRE against other state-of-the-art batch correction methods

**Fig. 3.**
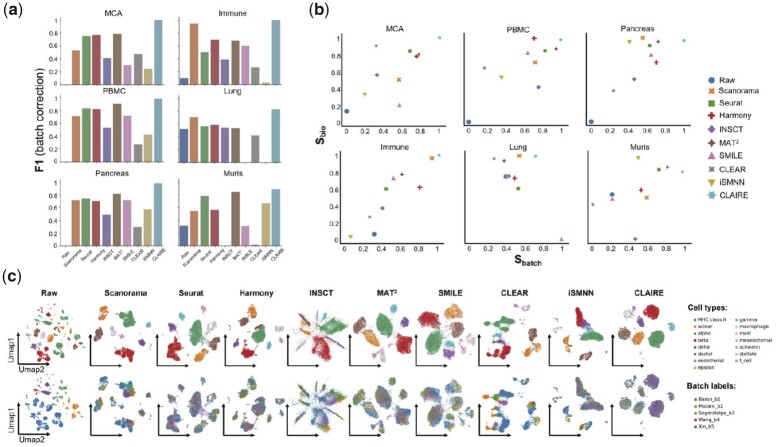
Benchmarking of CLAIRE against eight state-of-the-art batch correction methods. (**a**) F1 scores (batch correction) of $S_{\text{bio}}$ and $S_{\text{batch}}$ from nine methods. (**b**) $S_{\text{bio}}$ and $S_{\text{batch}}$ of nine methods on six datasets. (**c**) UMAP visualizations of nine methods’ outputs on Pancreas datasets

We benchmark CLAIRE against eight state-of-the-art batch correction methods on six real datasets. Three classical methods, Seurat, Scanorama and Harmony, and four CL-based methods, INSCT, MAT^2^, SMILE and CLEAR are included for comparison. In addition, we also include iSMNN (Yang *et al.*, 2021b) in our benchmarking, which performs iterative MNN refinement in a non-neural network style to facilitate sufficient batch correction. The main differences between iSMNN’s refinement strategy and CLAIRE’s are that iSMNN adds more MNNs within multiple iterations of MNN refinement while CLAIRE directly removes some MNNs within only one iteration. All competing methods’ settings are shown in Supplementary Note S1. The benchmarking results are displayed in Figure 3. Figure 3a shows that CLAIRE reaches the highest F-scores on all datasets, indicating that CLAIRE achieves the best trade-off between batch mixing and preservation of cellular heterogeneity. More specifically, CLAIRE reaches the highest batch mixing scores on all datasets except for Lung dataset and the top-2 heterogeneity conservation scores on five datasets (Fig. 3b and Supplementary Table S1). In particular, CLAIRE outperforms the second-best methods by 22% in terms of kBET on average on five datasets other than Lung dataset. Note that for Lung dataset, SMILE and INSCT are the only two methods that obtain higher kBET scores than CLAIRE while they greatly compromise to ARI and NMI (Supplementary Table S1).

To better illustrate CLAIRE’s performance, we visualize all methods’ outputs on six datasets by UMAP in Figure 3c and Supplementary Figures S3–S7. From the visualizations, it can be seen that CLAIRE retains clearly separable cell clusters and batches are mixed sufficiently within each cluster, which is consistent with the evaluation results. Particularly, on Pancreas dataset, batches are uniformly distributed within each cell type cluster in CLAIRE’s results while in other methods’ results, there is always a small part of ‘Baron_b1’ batch isolated in an area, which explains CLAIRE’s remarkable improvement of kBET on this dataset. Moreover, though adopting only one iteration of MNN refinement, CLAIRE achieves more sufficient batch mixing than iSMNN and there are less over-correction phenomena in its visualizations, which demonstrates the advantages of our proposed CL framework. What’s more, though CLAIRE accomplishes highly sufficient batch mixing results, it does not mix rare cell types with common cell types. For instance, on PBMC dataset, CLAIRE retains clearly separated clusters of hematopoietic stem cells and Megakaryocytes, epsilon cells in Pancreas dataset, and ionocytes cluster in Lung dataset.

Time and memory consumptions are important issues when evaluating batch correction methods. We evaluate CLAIRE and other batch correction methods with respect to their computation time and memory usage with a Linux server with 48-core Intel Xeon Silver 4116 CPU, 256 GB RAM and GeForce RTX 2080 Ti. We sample various number of cells from Tabular Muris Senis dataset (Tabula Muris Consortium, 2020) with range from 2000 to 120 000, and assess all methods on these sampled datasets. Time of reading data is not recorded for all methods. Evaluation results are shown in Supplementary Tables S2 and S3. From the results, it can be seen that CLAIRE’s time and memory consumptions both increase nearly linearly to the number of cells, and its overall consumption is comparable with other state-of-the-art methods.

### 3.5 Effectiveness of CLAIRE’s construction and refinement strategy

To validate the effectiveness of CLAIRE’s construction and refinement strategy, we conduct an ablation study. In particular, we compare CLAIRE with two variants: (i) CLAIRE without our proposed construction strategy, which follows INSCT and directly uses inter-batch MNNs as positive pairs and intra-batch KNNs as positive samples for those cells without MNN, denoted as CLAIRE-var1; and (ii) CLAIRE without refinement strategy, denoted as CLAIRE-var2. Each variant is run for five times on Pancreas dataset and Immune datasets, respectively. Ablation results are shown in Supplementary Figure S8a. We observe that the CLAIRE shows higher kBET scores than CLAIRE-var1 after convergence, which verifies that our construction strategy better covers the whole distributions of shared populations between batches. CLAIRE-var2 achieves similar batch mixing scores as CLAIRE and their ARI/NMI increase to similar values after several epochs. However, as training proceeds, CLAIRE-var2’s ARI and NMI continuously drop while CLAIRE’s ARI and NMI are almost unchanged. On Pancreas dataset, CLAIRE-var2’s final NMI drops 10% compared to the peak value, and on Immune dataset, its NMI drops 13% compared to the peak value. By visualizing CLAIRE-var2’s embeddings on Pancreas dataset at different epochs (Supplementary Fig. S8b), we find that CLAIRE-var2 gradually mixes some cell clusters of different cell types, which indicates that there exist number of false positive pairs leading to over-correction. Nevertheless, we observe that CLAIRE’s results do not show obvious decline of heterogeneity, which verifies the importance of refinement strategy for reducing the impact of false positive pairs. Overall, our proposed construction strategy and refinement strategy both are indispensable for CLAIRE’s superior performance.

**Fig. 4.**
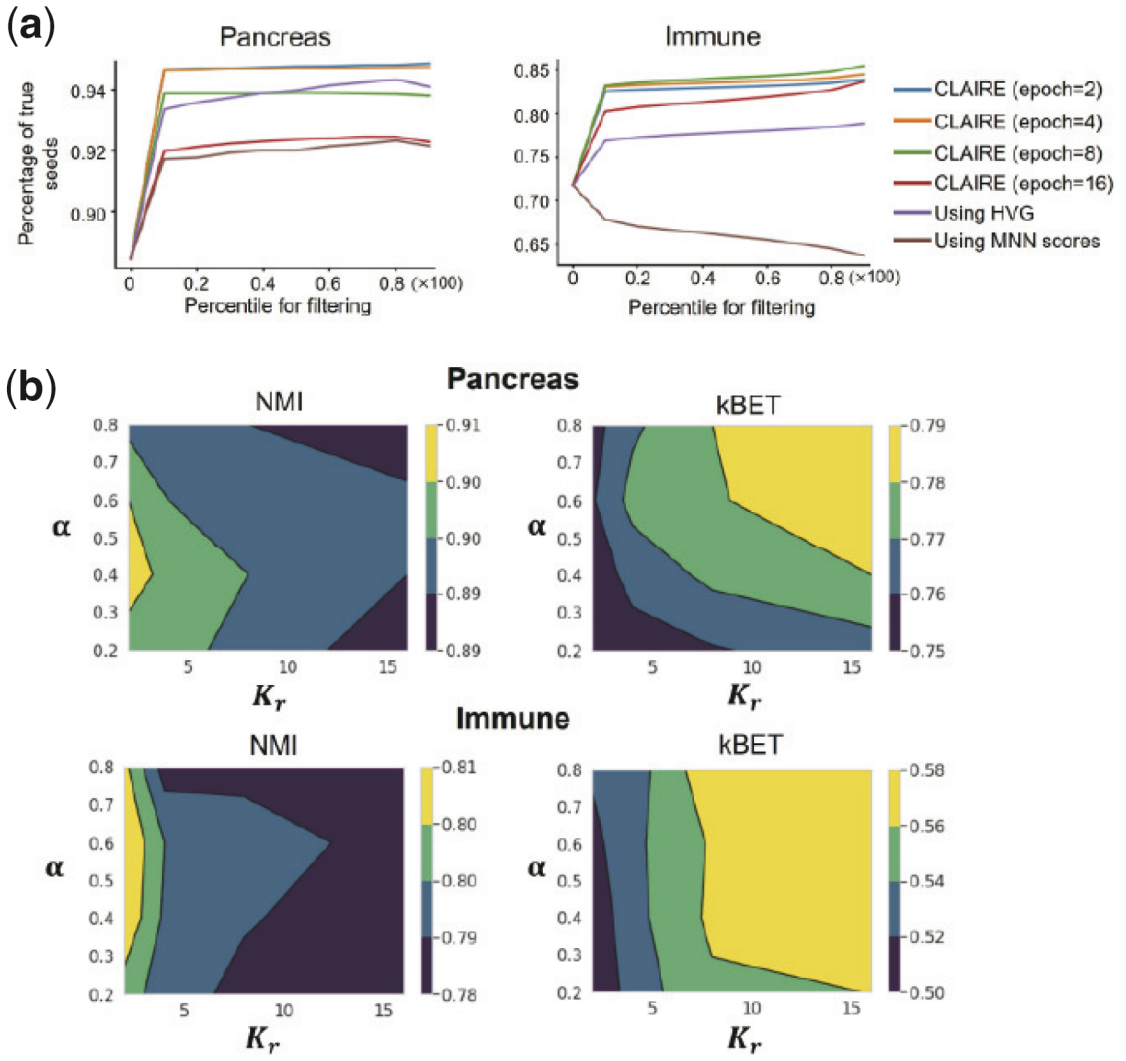
Ablation studies of CLAIRE on Pancreas and Immune datasets. (**a**) True seeds’ percentage of retained seeds obtained using three filtering approaches. ‘CLAIRE (epoch = 2)’ means filtering using CLAIRE’s embeddings after training 2 epochs. (**b**) CLAIRE’s NMI and kBET on Pancreas and Immune datasets by setting different α and $K_{r}$

To investigate whether two training stages are necessary for the refinement, we design two different approaches to filter seeds (inter-batch MNNs) directly based on the cellular expression profiles: (i) using HVG. After preprocessing dataset, calculating the cosine similarity between each seed pair using normalized expressions, and then building a two-component Gaussian mixture model on the similarities to infer false seeds. (ii) Using MNN scores. Following Seurat (Stuart *et al.*, 2019), we find the intra-batch KNN and inter-batch KNN for each cell. Each seed pair is scored by computing the overlap of their shared nearest neighbors. A Gaussian mixture model is built on the scores to infer false seeds. Figure 4a shows the true seeds’ percentage of retained seeds after filtering using three approaches. It can be seen that filtering on the learned representations leads to more correct seeds than other two approaches and training with 2–4 epochs generally lead the best results. Next, we apply the retained seeds (η = 0.2) from approach 1 for only one stage training. Interestingly, Supplementary Figure S9a shows that filtering using HVGs performs similarly with CLAIRE on Pancreas dataset while its performance drops on Immune dataset. We think their performance difference on Immune dataset is because CLAIRE obtains much higher percentage of true seeds. To study the generalizability of CLAIRE’s refinement strategy, we further evaluate three filtering approaches on other four datasets with respect to the true seeds’ percentage of retained seeds. Supplementary Figure S9b shows that CLAIRE outperforms the other two approaches on four datasets and shows significant improvements on complex datasets, such as Lung and Immune dataset, suggesting the superiority of our proposed refinement strategy.

We also explore the effect of hyper-parameters on two strategies, i.e. α and $K_{r}$ for the construction strategy, η for the refinement strategy. α refers to the parameter of uniform distribution during preparing positive pairs. $K_{r}$ refers to the number of nearest neighbors for searching intra-batch KNNs. η refers to the confidence threshold for filtering inter-batch MNNs. Results are shown in Figure 4b and Supplementary Figure S10. We find that smaller α and $K_{r}$ favor heterogeneity preservation scores while higher α and $K_{r}$ favor batch mixing scores. Overall, we find that $K_{r}=10$, α=0.5 achieve desirable results. Interestingly, we find that CLAIRE is insensitive to η. Supplementary Figure S10b shows that when η increases, all metrics show small changes, which indicates that CLAIRE infers true seeds with very high confidence and further demonstrates the robustness of our refinement strategy.

## 4 Discussion

We present a novel CL-based batch correction framework, CLAIRE, for integrating scRNA-seq datasets. The key idea is to improve the appropriateness of positive pairs which can dominate the results of batch correction. We propose two complementary strategies to realize appropriate positive pairs. First, to improve the coverage of positive pairs for the distribution of shared populations between batches, we propose a dynamical construction strategy for positive pairs by exploiting inter-batch MNN and intra-batch KNN. Our construction strategy not only helps positive pairs better cover the shared distributions between batches but is also computationally efficient. Second, to improve the correctness of positive pairs, we propose a refinement strategy to remove false positive pairs. Experiment results show that CLAIRE achieves superior mix-heterogeneity trade-off over existing CL-based batch correction methods. When benchmarking on six real datasets, CLAIRE outperforms eight state-of-the-art batch correction methods with respect to the best comprehensive performance of dataset integration.

We conduct extensive ablation experiments to verify the effectiveness of CLAIRE. Two variants of CLAIRE, CLAIRE-var1 and CLAIRE-var2, both show inferior performance than CLAIRE in terms of batch mixing score and heterogeneity preservation score. In particular, even with the refinement strategy, CLAIRE-var1’s final NMI values still show distinct drop compared to the initial peak values. The probable reason for the drop is that positive pairs defined by intra-batch NNs have inconsistent pattern with those defined by inter-batch MNNs, interfering the refinement process. These findings demonstrate that our proposed two strategies are indispensable for CLAIRE’s superior performance. Moreover, we show that two-stage training greatly helps to discriminate true seeds and false seeds, which promotes better refinement of positive pairs and validates the memorization effect of deep neural networks.

To further demonstrate the utility of CLAIRE for single-cell data analysis, we conduct various downstream analysis, including label transfer between scRNA-seq datasets, cross-omics label transfer, and trajectory analysis, based on CLAIRE’s outputs (Supplementary Note S2). Analysis results demonstrate that CLAIRE’s integrated embeddings can accurately transfer labels between scRNA-seq datasets and across omics. Additionally, CLAIRE can preserve the contiguous structure among cells after removing batch effects, which can facilitate further analysis about cell development.

Although CLAIRE achieves notable performance, there are still some limitations to be improved. One major limitation is that CLAIRE needs MNNs as signals to merge batches. However, computing MNNs between every pair of batches is time consuming, which is the major bottleneck of CLAIRE’s computation consumption. Some MNN-free batch correction methods, such as DESC (Li *et al.*, 2020), can also achieve robust correction performance. Therefore, we can combine ideas from those MNN-free methods with CL to realize more effective batch correction framework. Another limitation is that CLAIRE adopts Moco-style CL architecture, which relies on negative pairs to achieve robust representations. Recently, some negative-free CL methods have been proposed and they are more resilient than its counterparts in many aspects (Chen and He, 2021; Grill *et al.*, 2020), which can be adapted into CLAIRE’s framework. What’s more, considering CLAIRE’s excellent performance in integrating scRNA-seq datasets, we believe it has a great potential to migrate to multi-omics data integration problems.

## Supplementary Material

btad099_Supplementary_Data
