# Supplementary material for: CLAIRE: contrastive learning-based batch correction framework for better balance between batch mixing and preservation of cellular heterogeneity
Source: Bioinformatics. 2023 Feb 23;39(3):btad099. doi: 10.1093/bioinformatics/btad099 (PMC9985174; doi:10.1093/bioinformatics/btad099)
Supplement: btad099_Supplementary_Data [file btad099_supplementary_data.pdf]

SUPPLEMENTARY INFORMATION

CLAIRE: a novel contrastive learning-based batch correction framework for better balance between batch mixing and preservation of cellular heterogeneity.

Supplementary Tables ----------------2-3
Supplementary Notes ---------------4-6
Supplementary Figures ----------------7-12
Reference ----------------13

Supplementary Table S1. Detailed benchmarking results of eight methods.

Dataset	Metrics	Raw	Scanorama	Seurat	Harmony	INSCT	MAT^2	SMILE	CLEAR	iSMNN	CLAIRE
MCA	NMI	0.668	0.730	0.776	0.761	0.718	0.747	0.628	0.776	0.707	**0.786**
	ARI	0.337	0.495	0.669	0.657	0.581	0.716	0.518	0.725	0.400	**0.783**
	bASW	0.642	0.835	0.843	0.850	0.642	0.820	0.825	0.742	0.729	**0.910**
	kBET	0.003	0.186	0.274	0.331	0.295	0.401	0.207	0.122	0.033	**0.451**
	S_batch	0	0.565	0.679	0.753	0.327	0.777	0.569	0.320	0.195	**1**
	S_bio	0.125	0.502	0.843	0.781	0.557	0.802	0.203	0.903	0.319	**1**
	$F1_{bc}$	0	0.531	0.752	0.767	0.412	0.789	0.299	0.472	0.242	**1**
PBMC	NMI	0.648	0.811	0.838	**0.883**	0.753	0.839	0.825	0.793	0.784	0.873
	ARI	0.374	0.733	0.818	0.869	0.562	0.835	0.819	0.706	0.607	**0.873**
	bASW	0.656	0.937	0.895	0.910	0.835	**0.939**	0.892	0.745	0.821	0.932
	kBET	0.001	0.339	0.620	0.395	0.680	0.691	0.342	0.018	0.094	**0.784**
	S_batch	0	0.713	0.819	0.701	0.750	0.941	0.635	0.168	0.352	**0.988**
	S_bio	0	0.707	0.849	**0.995**	0.413	0.868	0.822	0.641	0.523	0.977
	$F1_{bc}$	0	0.710	0.833	0.823	0.532	0.903	0.716	0.266	0.420	**0.983**
Pancreas	NMI	0.644	**0.915**	0.878	0.814	0.778	0.895	0.835	0.748	0.891	0.900
	ARI	0.258	**0.951**	0.921	0.804	0.618	0.941	0.883	0.504	0.938	0.944
	bASW	0.529	0.848	0.818	0.883	0.706	0.841	0.868	0.700	0.748	**0.917**
	kBET	0	0.227	0.406	0.382	0.369	0.502	0.328	0.031	0.203	**0.783**
	S_batch	0	0.557	0.631	0.701	0.465	0.723	0.647	0.240	0.413	**1**
	S_bio	0	**1**	0.911	0.708	0.508	0.956	0.803	0.370	0.946	0.968
	$F1_{bc}$	0	0.715	0.746	0.705	0.485	0.823	0.716	0.291	0.575	**0.984**
Immune	NMI	0.630	0.787	0.715	0.711	0.678	0.743	0.735	0.653	0.609	**0.798**
	ARI	0.257	0.807	0.609	0.644	0.461	0.720	0.698	0.416	0.277	**0.816**
	bASW	0.809	0.899	0.793	0.893	0.730	0.812	0.882	0.785	0.653	**0.904**
	kBET	0.001	0.500	0.180	0.362	0.284	0.333	0.069	0.001	0.068	**0.576**
	S_batch	0.310	0.924	0.435	0.793	0.399	0.605	0.515	0.262	0.059	**1**
	S_bio	0.056	0.962	0.596	0.617	0.366	0.770	0.727	0.260	0.018	**1**
	$F1_{bc}$	0.095	0.943	0.503	0.694	0.382	0.678	0.603	0.261	0.027	**1**
Lung	NMI	0.697	0.715	0.528	0.633	0.625	0.705	0.190	**0.720**	NaN	**0.720**
	ARI	0.362	**0.586**	0.376	0.400	0.433	0.535	0.098	0.546	NaN	0.576
	bASW	0.863	0.913	0.802	0.854	0.665	0.815	**0.914**	0.797	NaN	0.856
	kBET	0	0.019	0.103	0.045	0.174	0.031	**0.203**	0.001	NaN	0.136
	S_batch	0.398	0.544	0.528	0.491	0.429	0.378	**1**	0.269	NaN	0.719
	S_bio	0.748	**0.995**	0.604	0.727	0.753	0.934	0	0.959	NaN	0.989
	$F1_{bc}$	0.519	0.704	0.564	0.586	0.546	0.538	0	0.420	NaN	**0.832**
Muris	NMI	0.789	0.790	0.808	0.794	0.761	0.804	0.789	0.788	**0.818**	0.796
	ARI	0.650	0.638	0.684	0.654	0.577	0.700	0.638	0.622	0.695	**0.704**
	bASW	0.775	0.853	0.828	0.805	0.687	0.855	0.762	0.670	0.787	**0.907**
	kBET	0.009	0.314	0.573	0.366	0.626	0.628	0.051	0.020	0.378	**0.708**
	S_batch	0.222	0.604	0.737	0.541	0.477	0.833	0.224	0.008	0.511	**1**
	S_bio	0.533	0.495	0.834	0.588	0	0.861	0.486	0.414	**0.965**	0.807
	$F1_{bc}$	0.313	0.544	0.782	0.563	0	0.847	0.307	0.015	0.668	**0.893**

NaN means we cannot get the method to execute properly on this dataset. The detail can be found in Supplementary Note 1.

Supplementary Table S2. Computational consumptions of nine methods.

	Cells	Scanorama	Seurat	Harmony	INSCT	MAT^2	SMILE	CLEAR	iSMNN	CLAIRE
Time(sec)	2000	8.01	17.94	2.80	59.73	49.60	15.57	147.46	NaN	48.49
	4000	15.72	22.40	7.79	109	68.90	21.56	251.09	NaN	80.21
	8000	32.69	37.04	16.16	201	103.86	41.44	472.84	108	127.62
	16000	72.61	78.60	31.78	386.26	165.89	80.47	918.83	120	226.71
	30000	150.37	220.80	55.39	738.76	353.25	96.71	1694.14	336	506.14
	60000	351.47	739.80	128.93	1482	901.59	201.27	3333.19	450	1166.18
	120000	906.61	2400.00	305.4	2947.72	2733.76	391.63	6650.64	1086	3324.38
Memory(Mb)	2000	902.80	547.90	575.1	811.6	4290.20	4109.90	4413.20	NaN	3339.9
	4000	1001.60	653.80	701.5	886	4399.80	4178.80	4419.60	NaN	3364.1
	8000	1079.30	866.10	955.1	1053	4477.40	4089.40	4437.30	993	3440.8
	16000	1354.90	1288.30	1462.4	1285.3	4602.20	4109.90	4469.30	1542	3620.7
	30000	1770.90	2025.40	2350.2	1618.7	4979.30	4153.80	4509.40	2500	3941.9
	60000	2604.70	3612.00	4251.5	2570.4	5721.80	4169.50	4547.10	4559	4557
	120000	4306.70	6790.70	8055.4	4631.9	7128.70	4286.10	4688.10	8679	5658

Supplementary Table S3. Time consumption (seconds) of five steps in CIAIRE.

Cells	Step 1	Step 2	Step 3	Step 4	Step 5	Total time
2000	10	27.52	0.89	9.27	0.81	48.49
4000	21.6	37.23	1.15	19.12	1.11	80.21
8000	29.6	55.35	1.72	39.41	1.54	127.62
16000	61.92	83.41	2.53	76.32	2.53	226.71
30000	182.4	160.88	4.83	153.02	5.01	506.14
60000	576	273.79	8.57	299.35	8.47	1166.18
120000	2160	530.55	16.54	600	17.29	3324.38

Step 1: finding mutual nearest neighbors (MNN) between batches; Step 2: dynamic generation of positive pairs during training; Step 3: filtering (MNN) pairs during training; Step 4: model forward and backpropagation during training; Step 5: model inference.

Supplementary Table S4. CLAIRE's accuracy of label transfer between scRNA-seq datasets.

Dataset	Scanorama	Seurat	Harmony	CLEAR	INSCT	MAT^2	SMILE	CLAIRE
MouseCellAtlas	**0.870**	<u>0.854</u>	0.839	0.817	0.765	0.797	0.679	0.850
PBMC	0.970	**0.972**	0.964	0.938	0.870	0.950	0.943	<u>0.970</u>
Immune Human	<u>0.881</u>	0.822	0.759	0.816	0.642	0.827	0.829	**0.894**
Lung	<u>0.636</u>	0.568	0.596	0.600	0.512	0.580	0.265	**0.650**

Supplementary Note 1

We benchmarked the performance of CLAIRE with eight methods designed to integrate scRNA-seq datasets: Scanorama, Seurat (v.3), Harmony, INSCT, MAT^2, SMILE, CLEAR, and iSMNN. Experimental settings used for each method are as follows:

- Scanorama: Python package Scanorama v.1.7.1 was used for all the datasets. All datasets were preprocessed as described in the manuscript. Then, following the practice of Luecken et al. [1], we scaled the data matrix of each batch separately before input to Scanorama. For all datasets, scanorama.correct_scanpy function was used for integration with default parameters. Finally, the integrated low-dimensional embeddings (adata.obsm['X_scanorama'], 100 dimensions) were used for evaluation.
- Seurat: R package Seurat v.3.2.2 was used for all datasets. The raw count matrix of scRNA-seq was used as input. For each dataset, low informative genes expressed in fewer than three cells were filtered. Then, each dataset was split into multiple batches using SplitObject function. Each batch was normalized using NormalizeData function and top 2000 most highly variable genes (top 5000 for the Muris dataset) were selected for each batch using FindVariableFeatures function with vst method. SelectIntegrationFeatures function was used to combine features from all batches and these selected features were used as 'anchor.features' in FindIntegrationAnchors function to find anchors between batches. Those found anchors were used as 'anchorset' in IntegrateData function to integrate all batches.
- Harmony: R package harmony v0.1 and Seurat v3.2.2 were used for all datasets. The raw count matrix of scRNA-seq was used as input. For each dataset, low informative genes expressed in fewer than three cells were filtered. Then, each dataset was split into multiple batches using Seurat::SplitObject function. Each batch was normalized using Seurat::NormalizeData function and top 2000 most highly variable genes (top 5000 for the Muris dataset) were selected for each batch using Seurat::FindVariableFeatures function with vst method. Then, data was scaled using Seurat::ScaleData function. PCA was performed using Seurat::RunPCA function with 30 principal components (pcs). Finally, harmony::RunHarmony function was performed on the 30 pcs for integration.
- INSCT: Python package insct was used for all datasets. All datasets were preprocessed as described in our manuscript. PCA was performed on scaled data with 50 principal components. Preprocessed dataset and 50 principle components were used as input of INSCT. insct.tnn.tnn.TNN.fit and insct.tnn.tnn.TNN.transform functions with default parameters were used to integrate all datasets, which produced batch corrected 2-dimensional embeddings.
- MAT^2: Python package MAT^2 was used for all datasets. All datasets were preprocessed as described in our manuscript. MAT^2 needs precomputed anchors between batches for integration. We used the anchors exported from Seurat::FindIntegrationAnchors function for MAT^2. Preprocessed dataset and inter-batch anchors were used as input for MAT^2. MAT2.BuildMAT2.train function and MAT2.BuildMAT2.evaluate function were used to integrate preprocessed datasets, which produced batch corrected low-dimensional embeddings. Training and inference both used default parameters.
- SMILE: Python package SMILE was used for all datasets. All datasets were preprocessed as described in our manuscript. SMILE further standardized features of each dataset by removing the mean and scaling to unit variance. SMILE.SMILE.SMILE_trainer function was used to integrate the preprocessed datasets with default parameters. Then, the preprocessed datasets were input to the encoder network in mini batch (due to batch normalization layer in the network) to obtain batch corrected low-dimensional embeddings.
- CLEAR: Python package CLEAR was used for all datasets. CLEAR script provided many options for preprocessing, such as filtering, log-normalization, selection of highly variable genes, and so on. Through our experiments, we found that using log-normalization and selection of HVGs produced consistently good results. Preprocessed dataset was saved in .h5ad format and its path was used as the parameter '—input_h5ad_path' when running CLEAR.py in terminal. Other parameters were as default.
- iSMNN: R package iSMNN was used for all datasets. iSMNN exploits cell type annotations and performs iterative mutual nearest neighbors refinement to improve MNNCorrect [2]. If cell type annotations unknown, iSMNN clusters each batch separately and annotates clusters using known marker genes. In our experiment, the ground truth cell type annotations were used as input of iSMNN. Each dataset was preprocessed sequentially using Seurat::SplitObject,

Seurat::NormalizeData, Seurat::FindVariableFeatures (top 2000 by default, top 5000 for Muris dataset). Then, preprocessed datasets and cell type annotations were used as input for iSMNN function with 'Short.run' strategy and 5 iterations. Other parameters were as default. Note that iSMNN requires at least one shared cell type among all batches and the minimum number of cells with a shared cell type in any batch should be greater than 'k.anchor' (default=5). In Lung dataset, there exists multiple shared cell types among all batches but the number of cells with a shared cell type in some batches is lower than 5, which will cause running errors. We tried to turn down the 'k.anchor' parameter but found it would cause other issues.

Supplementary Note 2

A. Label transfer between scRNA-seq datasets

Label transfer means leveraging information from annotated datasets (reference datasets) to automatically annotate cells in those datasets without cell type annotations (query datasets). Based on CLAIRE's integrated low-dimensional embeddings, k-nearest neighbors (kNN) classifier can be built between reference datasets and query datasets to infer the types of query cells. Other competing methods can also use kNN classifier to transfer labels. We included seven competing methods including Scanorama, Seurat, Harmony, INSCT, MAT^2, SMILE, and CLEAR for a comparison. The evaluation metric is accuracy which is computed on the common cell types between reference datasets and query datasets. We set the k of kNN classifier to 10 for all methods.

MouseCellAtlas, PBMC, Immune Human, and Lung datasets were used for experiments. Details of four datasets can be found in Table 1 from our manuscript. For MouseCellAtlas and PBMC datasets, label transfer is performed from 'Batch 1' to 'Batch 2' and from 'Batch 2' to 'Batch 1'. Average accuracy of two transfer tasks is used as the results for these two datasets. For Immune Human datasets, batch 'Oetjen_A' contains the most diverse cell types (16, 16 in total), which is used as reference datasets and the other batches are used as query datasets. For Lung dataset, batch '5' contains the most diverse cell types (15, 17 in total), which is used as reference dataset and the other batches are used as query datasets. Supplementary Table S4 showed the benchmarking results. It can be observed that CLAIRE achieved accurate label transfer on the first three datasets and reached the highest accuracy in two out of four datasets.

B. Cross-omics label transfer

CLAIRE can transfer cell type information between scRNA-seq and scATAC-seq. PBMC multiome dataset[1] was used for experiment. In this dataset, scRNA-seq and scATAC-seq profiles were simultaneously collected in the same cells. We treated the datasets as originating from two different experiments. As CLAIRE needs matched input features between datasets, we generated a rough estimate of the transcriptional activity of each gene by quantifying ATAC-seq counts in the 2 kb-upstream region and gene body, using the GeneActivity function in the Signac (v.1.7.0) [3] package. There are 19 cell types in total. The number of total cells is 20824 (10412+10412) and the number of common genes between two modalities is 18353. Two label transfer tasks are defined on this dataset: transfer labels from RNA to ATAC and transfer labels from ATAC to RNA. Accuracy is used as the evaluation metric.

We performed the same preprocessing steps as described in our manuscript and fed the preprocessed dataset to CLAIRE for training and inference. On the integrated low-dimensional embeddings, kNN classifier was used to infer cell types of query cells. For a comparison, MAT^2 was also tested on this dataset and its integrated data matrix was used to transfer labels using kNN classifier. We set the k of kNN classifier to 10 for both methods. For RNA->ATAC transfer task, MAT^2 achieved 76% accuracy and CLAIRE achieved 85% accuracy. For ATAC->RNA transfer task, MAT^2 achieved 77% accuracy and CLAIRE achieved 86% accuracy. We visualized the integration results via UMAP in Supplementary Figure S12. It can be observed that CLAIRE achieved more sufficient omics mixing than MAT^2 and cellular heterogeneity in CLAIRE's results was clearer, which well explained CLAIRE's higher accuracy. Overall, these results demonstrated that CLAIRE can accurately transfer cell type annotations across scRNA-seq and scATAC-seq.

[1] https://support.10xgenomics.com/single-cell-multiome-atac-gex/datasets/1.0.0/pbmc_granulocyte_sorted_10k

C. Trajectory analysis

We collected two mouse neocortex datasets from Du et al. [4]. The first dataset (dataset A) contains 10261 cortical cells from E10.5, E12.5, E14.5, E16.5 and E18.5 mouse embryos and the second dataset (dataset B) contains 6390 cortical cells from E11.5, E13.5, E15.5 and E17.5 mouse embryos. Biological variations are confounded by batch effect in raw data as shown in Supplementary Figure S13. The inferred diffusion pseudotime is ambiguous to understand. We applied CLAIRE to integrate these two datasets. Supplementary Figure S13 showed the integration results. It can be observed that CLAIRE successfully removed batch effect while preserving the heterogeneity between different cell states. Further, we evaluated whether CLAIRE preserved the contiguous structure of cells. Specifically, Scanpy's diffusion pseudotime [5] function was used for the integrated data, raw dataset A, and raw dataset B to infer their pseudotime, respectively. Following Ruan et al. [6], the NEC cells were specified as root cells for pseudotime inference. Then, we computed spearman correlation between the pseudotime of raw dataset A and its corresponding part in the integrated data. Correlation for dataset B was also computed. CLAIRE achieved correlation coefficients of 0.91 and 0.93 on dataset A and dataset B respectively, which suggested that CLAIRE can well preserves the contiguous structure of original data after batch correction.

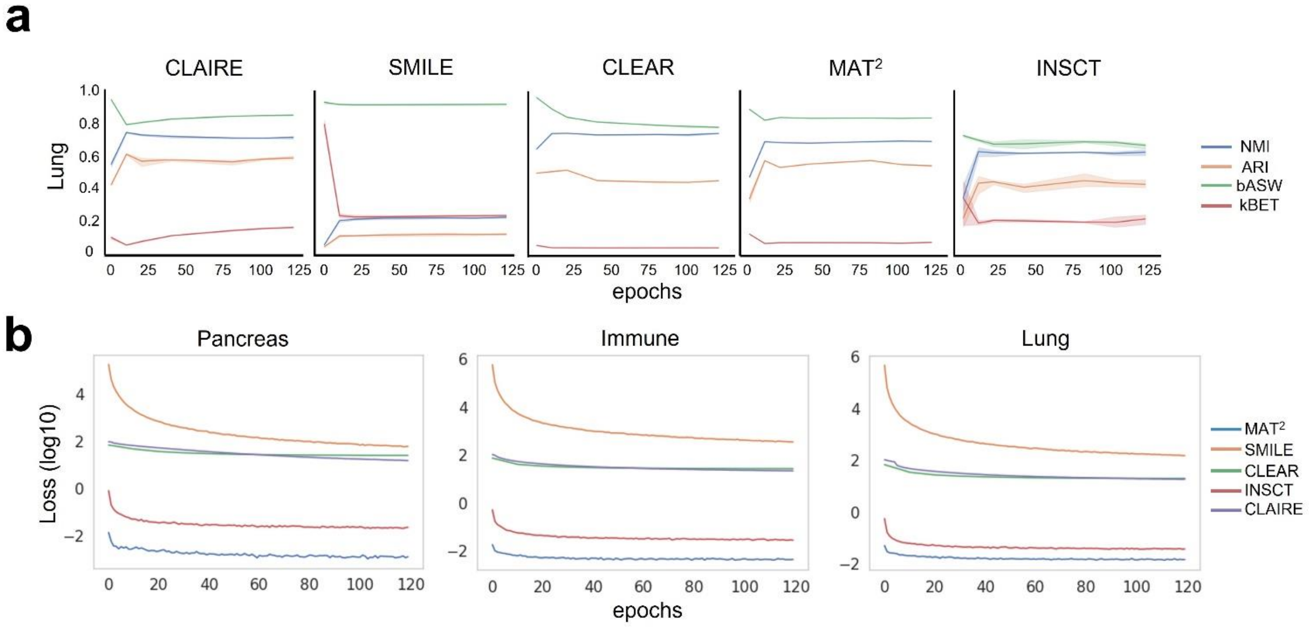

Fig. S1. Mixing-heterogeneity trade-off of contrastive learning-based batch correction methods. **(a)** Curves of four evaluation metrics with training epochs from CLAIRE, SMILE, CLEAR, MAT^2, INSCT on Lung datasets. **(b)** Training loss curves of five methods.

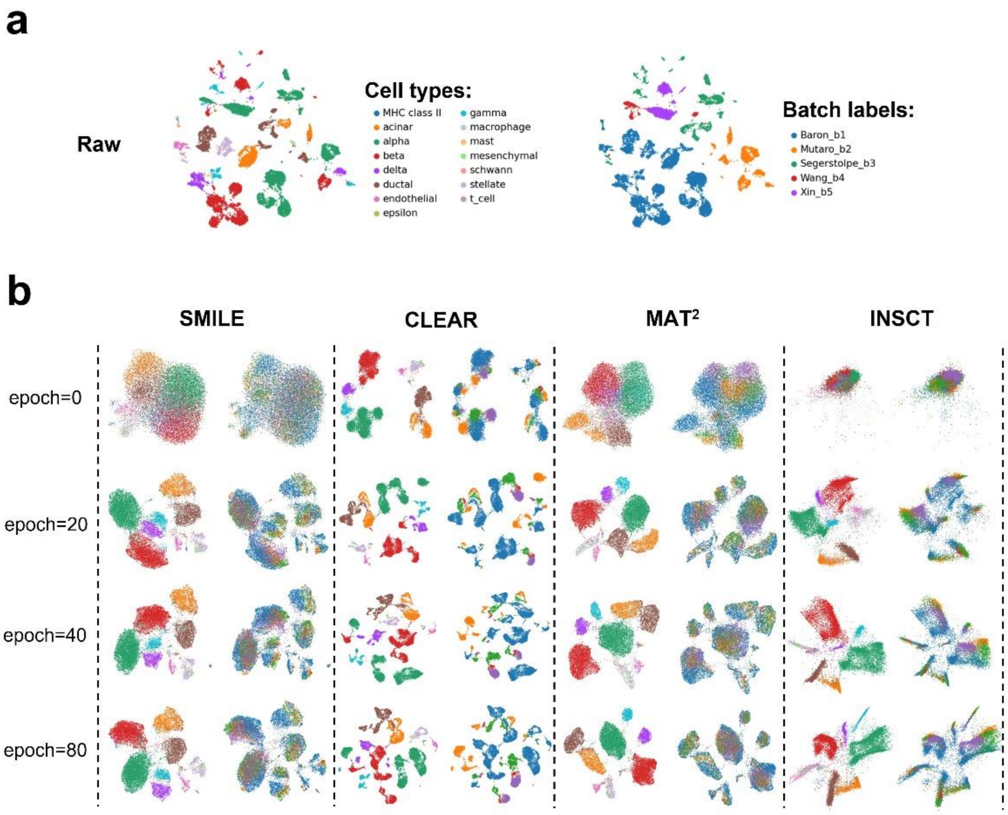

Fig. S2. UMAP visualizations of raw expression of Pancreas dataset and batch-corrected latent representations from SMILE, CLEAR, MAT^2, and INSCT. **(a)** Visualizations of raw expressions. **(b)** Visualizations of latent representations from four CL-based methods at different epochs. For each method, cells are colored by cell type in the first columns and colored by batch labels in the second column. The cell color markers are consistent with **a**.

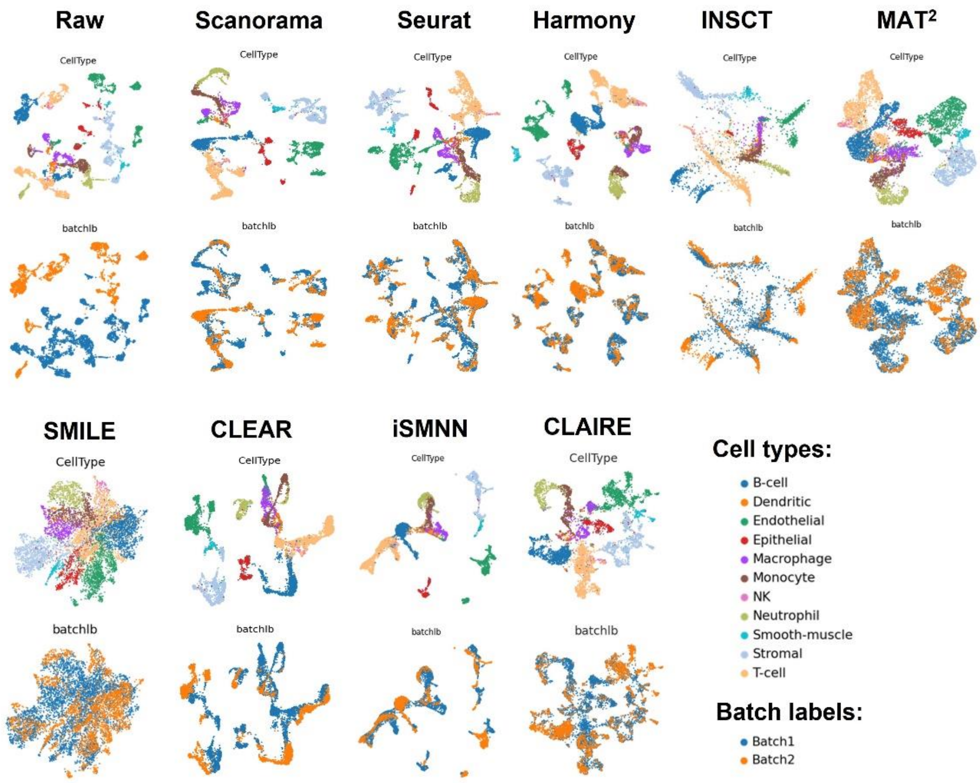

Fig. S3. UMAP visualizations of raw expression, and outputs from nine batch correction methods on MCA dataset. Each method's visualizations contain two rows. In the first row, cells are colored by cell types and colored by batch labels in the second row.

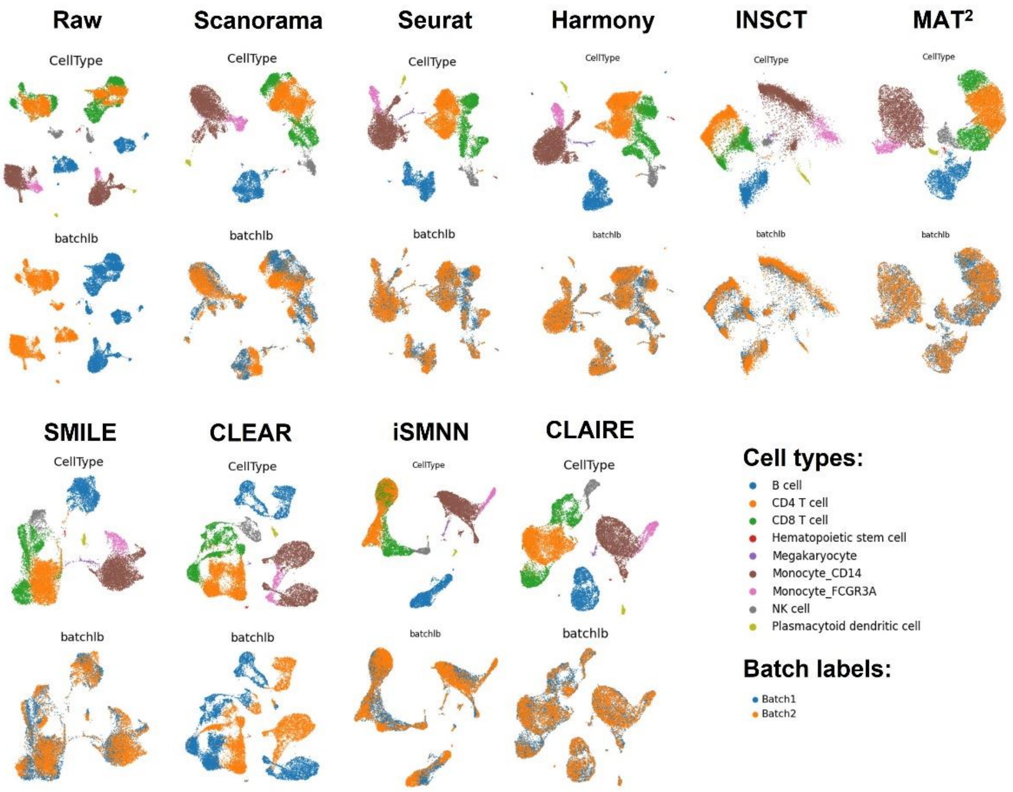

Fig. S4. UMAP visualizations of raw expression, and outputs from nine batch correction methods on PBMC dataset. Each method's visualizations contain two rows. In the first row, cells are colored by cell types and colored by batch labels in the second row.

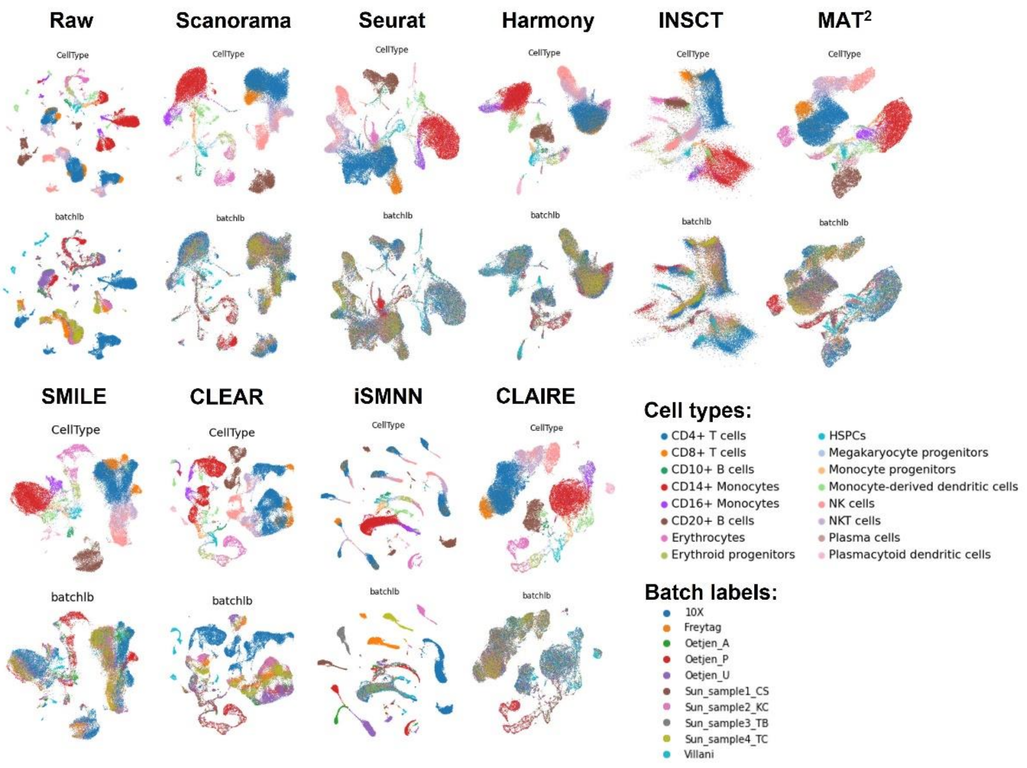

Fig. S5. UMAP visualizations of raw expression, and outputs from nine batch correction methods on Immune dataset. Each method's visualizations contain two rows. In the first row, cells are colored by cell types and colored by batch labels in the second row.

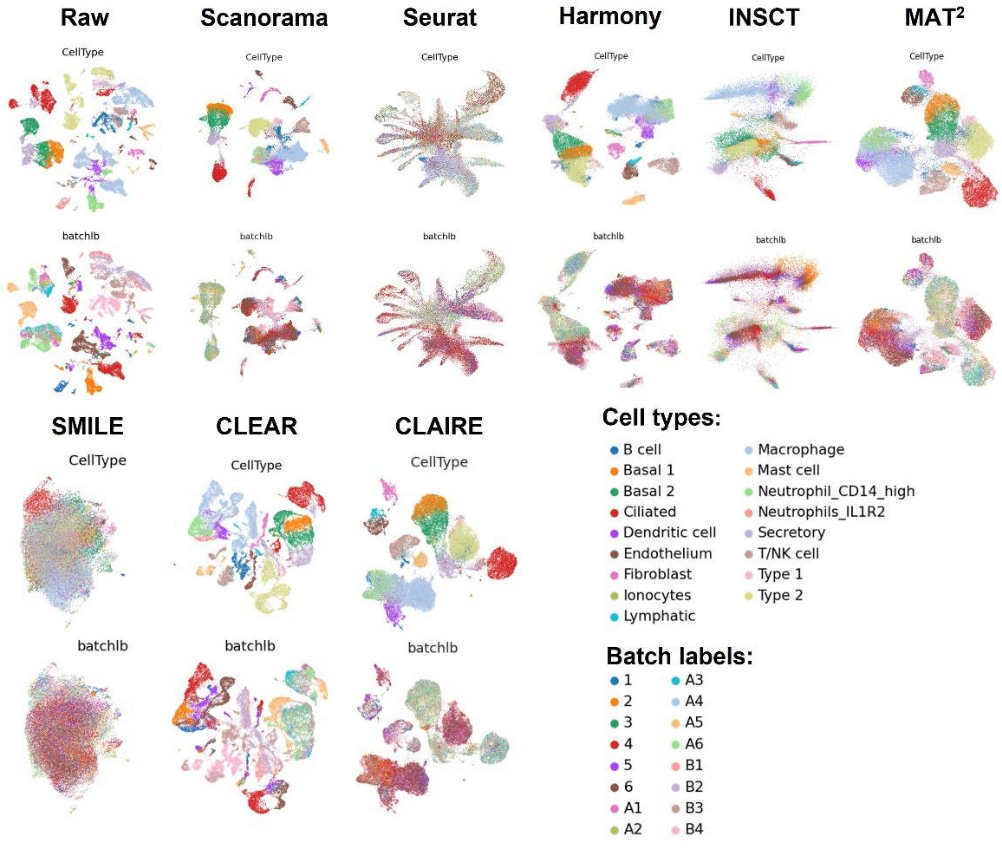

Fig. S6. UMAP visualizations of raw expression, and outputs from nine batch correction methods on Lung dataset. Each method's visualizations contain two rows. In the first row, cells are colored by cell types and colored by batch labels in the second row.

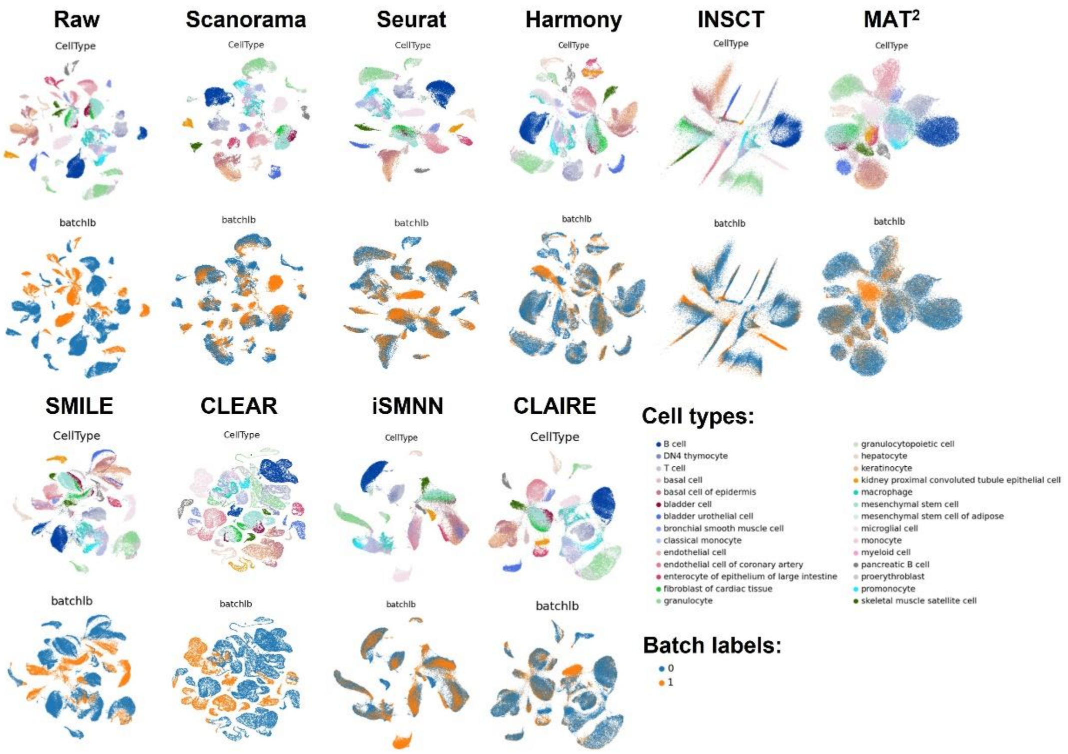

Fig. S7. UMAP visualizations of raw expression, and outputs from eight batch correction methods on Muris dataset. Each method's visualizations contain two rows. In the first row, cells are colored by cell types and colored by batch labels in the second row.

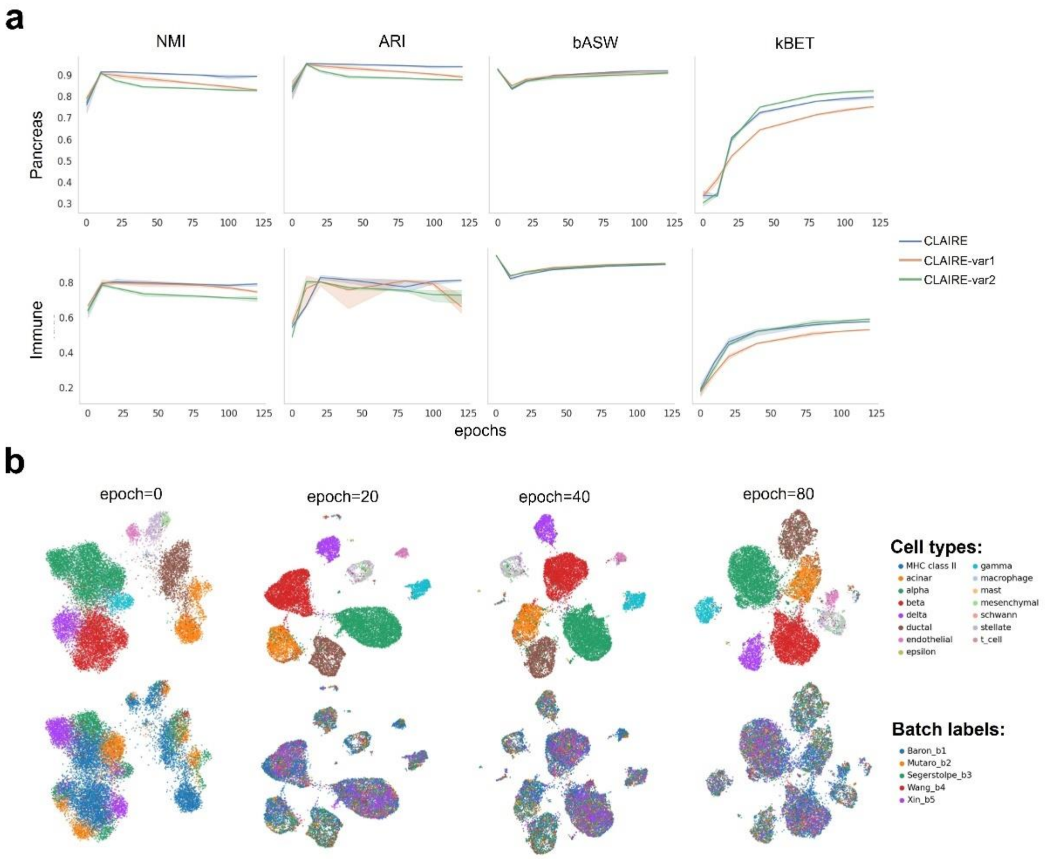

Fig. S8. Ablation studies for CLAIRE's two strategies. **(a)** Curves of four evaluation metrics with training epochs from CLAIRE, and its two variants on Pancreas and Immune datasets. **(b)** UMAP visualizations of CLAIRE-var2's embeddings at different epochs.

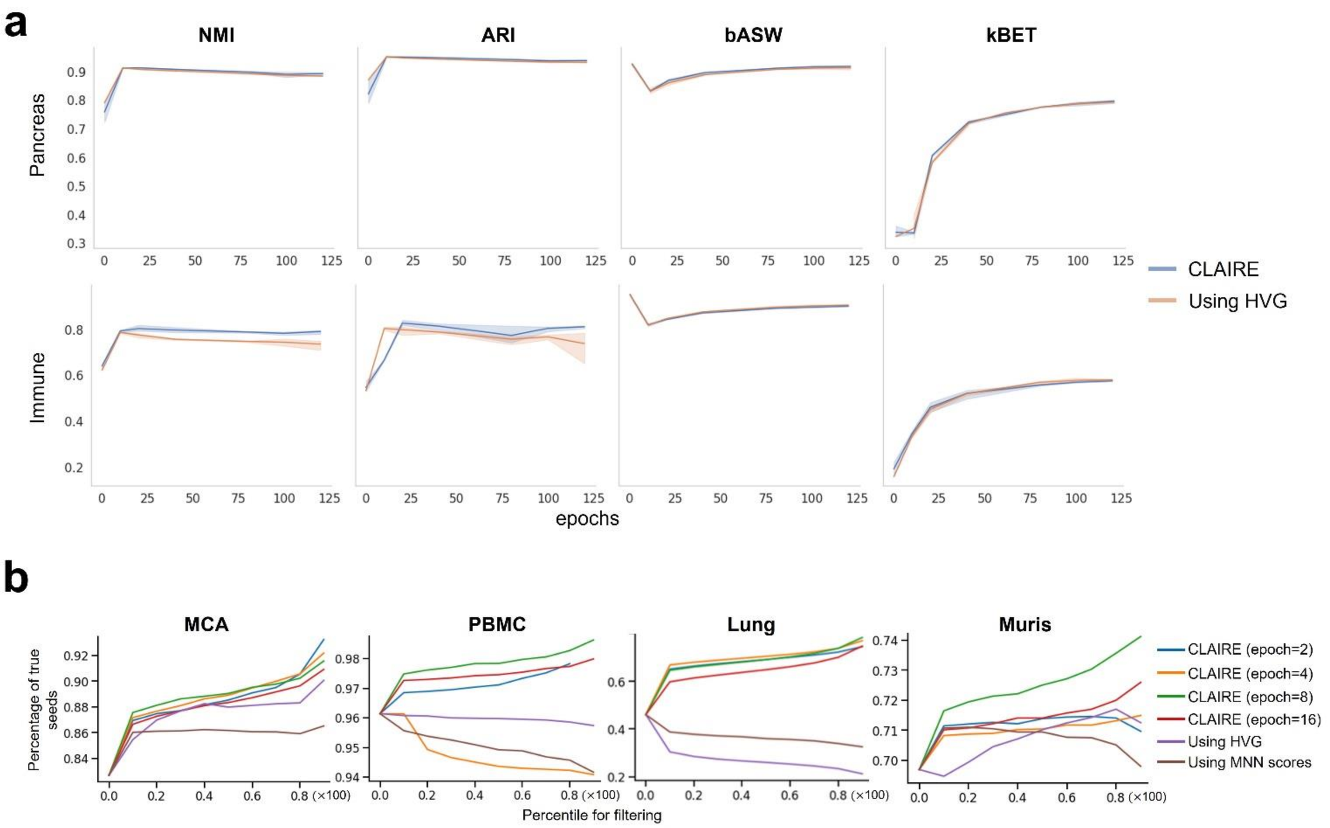

Fig. S9. Comparison of three approaches for filtering false seeds. **(a)** Curves of evaluation metrics produced by two filtering approaches on Pancreas and Immune datasets. **(b)** True seeds' percentage obtained using three filtering approaches on MCA, PBMC, Lung, and Muris datasets.

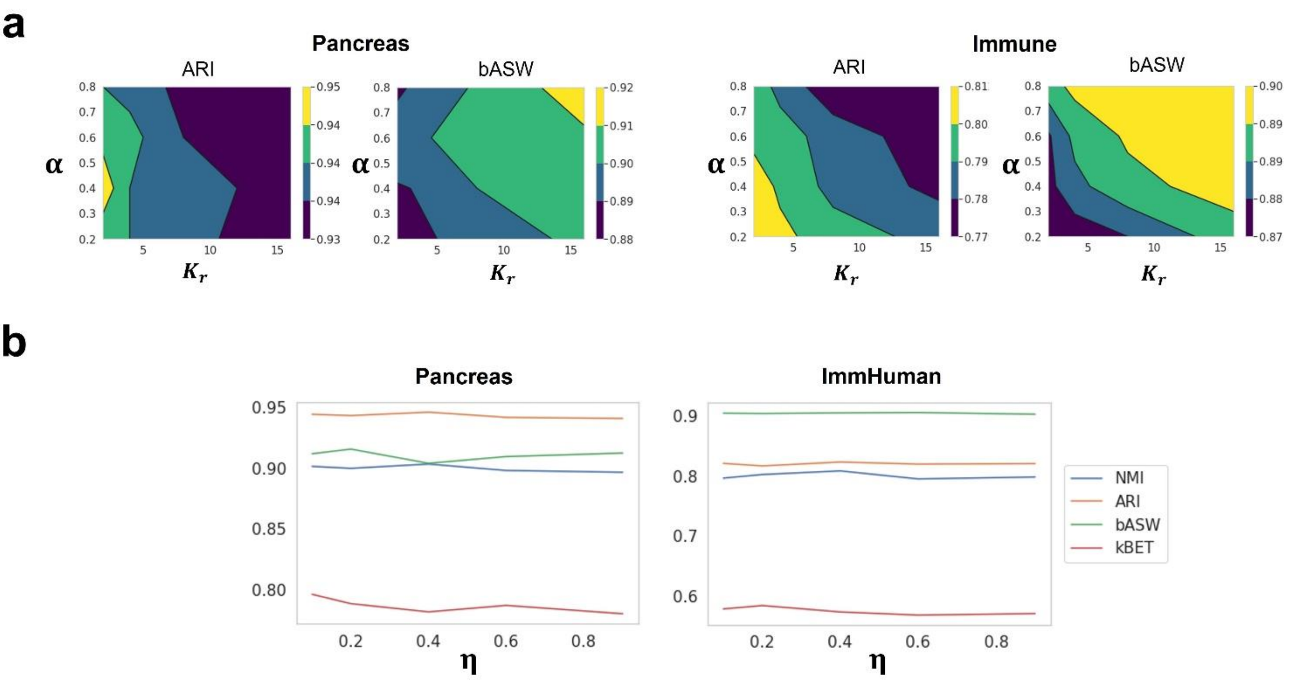

Fig. S10. Parameter experiments for α, K_r, and η. **(a)** CLAIRE's ARI and NMI on Pancreas and Immune datasets by setting different α and K_r. **(b)** CLAIRE's NMI, ARI, bASW, and kBET with different η settings.

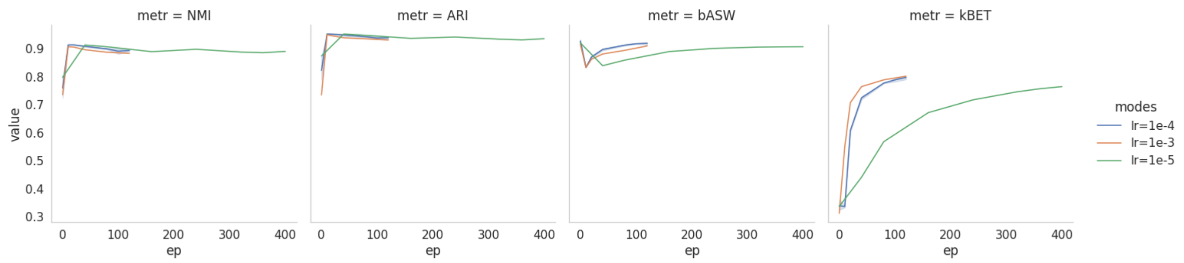

Fig. S11. Ablation study for learning rates on Pancreas dataset. ‘lr=1e-4’ is the default setting. When learning rate is smaller (1e-5), longer training epochs are needed for batch correction metrics to converge. Larger learning rate (lr=1e-3) performs similarly with lr=1e-4.

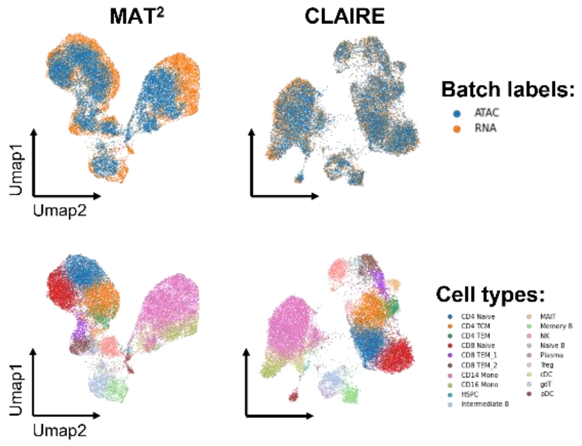

Fig. S12. UMAP visualizations of integrated latent representations from MAT^2 and CLAIRE. Cells are colored by omics in the first row and colored by cell types in the second row.

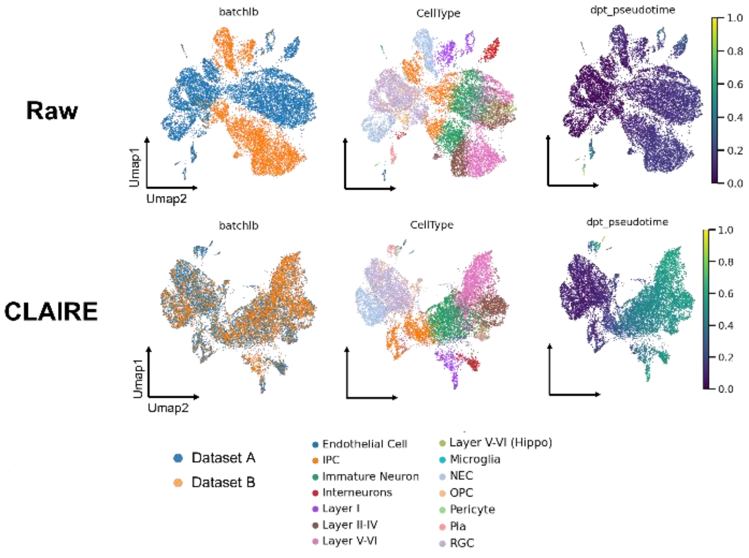

Fig. S13. UMAP visualization of mouse neocortex datasets using raw data and integrated data from CLAIRE. Cells are colored by batch labels in the first column, colored by cell types in the second column, and colored by diffusion pseudotime in the third column.

Reference

[1] Luecken, Malte D., et al. "Benchmarking atlas-level data integration in single-cell genomics." Nature methods 19.1 (2022): 41-50.

[2] Haghverdi, Laleh, et al. "Batch effects in single-cell RNA-sequencing data are corrected by matching mutual nearest neighbors." Nature biotechnology 36.5 (2018): 421-427.

[3] Stuart, Tim, et al. "Single-cell chromatin state analysis with Signac." Nature methods 18.11 (2021): 1333-1341.

[4] Du, Jin-Hong, Ming Gao, and Jingshu Wang. "Model-based trajectory inference for single-cell RNA sequencing using deep learning with a mixture prior." bioRxiv (2020).

[5] Wolf, F. Alexander, Philipp Angerer, and Fabian J. Theis. "SCANPY: large-scale single-cell gene expression data analysis." Genome biology 19.1 (2018): 1-5.

[6] Ruan, Xiangbin, et al. "Progenitor cell diversity in the developing mouse neocortex." Proceedings of the National Academy of Sciences 118.10 (2021): e2018866118.
